# App-Based Feedback for Rehabilitation Exercise Correction in Patients With Knee or Hip Osteoarthritis: Prospective Cohort Study

**DOI:** 10.2196/26658

**Published:** 2021-07-13

**Authors:** Johanna Theresia Biebl, Marzena Rykala, Maximilian Strobel, Pawandeep Kaur Bollinger, Bernhard Ulm, Eduard Kraft, Stephan Huber, Andreas Lorenz

**Affiliations:** 1 Department of Orthopaedics, Physical Medicine, and Rehabilitation University Hospital Ludwig Maximilians University of Munich Munich Germany; 2 Kaia Health GmbH Munich Germany; 3 Unabhängige statistische Beratung Bernhard Ulm Munich Germany

**Keywords:** mHealth, digital health, digital rehabilitation, machine learning, smartphone, osteoarthritis, exercise therapy

## Abstract

**Background:**

The use of digital therapeutic solutions for rehabilitation of conditions such as osteoarthritis provides scalable access to rehabilitation. Few validated technological solutions exist to ensure supervision of users while they exercise at home. Motion Coach (Kaia Health GmbH) provides audiovisual feedback on exercise execution in real time on conventional smartphones.

**Objective:**

We hypothesized that the interrater agreement between physiotherapists and Motion Coach would be noninferior to physiotherapists’ interrater agreement for exercise evaluations in a cohort with osteoarthritis.

**Methods:**

Patients diagnosed with osteoarthritis of the knee or hip were recruited at a university hospital to perform a set of 6 exercises. Agreement between Motion Coach and 2 physiotherapists’ corrections for segments of the exercises were compared using Cohen κ and percent agreement.

**Results:**

Participants (n=24) were enrolled and evaluated. There were no significant differences between interrater agreements (Motion Coach app vs physiotherapists: percent agreement 0.828; physiotherapist 1 vs physiotherapist 2: percent agreement 0.833; *P*<.001). Age (70 years or under, older than 70 years), gender (male, female), or BMI (30 kg/m^2^ or under, greater than 30 kg/m^2^) subgroup analysis revealed no detectable difference in interrater agreement. There was no detectable difference in levels of interrater agreement between Motion Coach vs physiotherapists and between physiotherapists in any of the 6 exercises.

**Conclusions:**

The results demonstrated that Motion Coach is noninferior to physiotherapist evaluations. Interrater agreement did not differ between 2 physiotherapists or between physiotherapists and the Motion Coach app. This finding was valid for all investigated exercises and subgroups. These results confirm the ability of Motion Coach to detect user form during exercise and provide valid feedback to users with musculoskeletal disorders.

## Introduction

Musculoskeletal conditions such as osteoarthritis and back pain result in a huge burden for patients and health care systems. Impaired mobility affects both the quality of life of the individual, for example, by increasing social isolation, and the health care system, by raising costs due to factors such as hospitalizations and secondary diseases [1-3]. Osteoarthritis can lead to pain-related fear of movement and an increased probability of further functional impairment [4]. In addition, osteoarthritis is a predictor for developing disabilities that affect activities of daily living, underlining the importance of effective interventions [5].

Current guidelines [6] recommend self-management programs and exercise as first-line therapies for managing osteoarthritis. The prevalence of osteoarthritis is increasing, yet cost and resource constraints limit in-person access to these therapies [7]. Digital therapeutics have emerged as an option to provide access to exercise therapy and multidisciplinary rehabilitation for patients with musculoskeletal pain conditions such as osteoarthritis and back pain [8-10]. Even though a recent survey among health professionals indicated widespread support of use of mobile health technologies in osteoarthritis treatment [11], a primary concern with using digital therapeutics for home-based exercise is the lack of supervision by health care professionals.

Several different digital solutions have been proposed to correct and optimize body pose during exercise execution to improve access to therapeutic exercises [12]. Many mobile health apps for musculoskeletal rehabilitation rely upon video instructions only and provide no means of detecting and correcting pose during exercise [9,13]. These systems, by default, leave users exposed to the risk of incorrectly performing exercise but allow for scalable access without requiring external hardware. To the best of our knowledge, there are no reports on the quality of exercise execution during the use of these systems. Other technologies, such as integrated devices containing inertial sensors, have also been validated to a limited extent, and whether they are suitable for detecting and correcting form during therapeutic exercises has not been evaluated [14,15]. Digital therapeutics that have been validated for this purpose require additional hardware such as a Microsoft Kinect device [16,17].

Motion Coach (Kaia Health GmbH) was recently introduced to address these issues (ie, requiring that equipment be worn on the body or additional hardware) by using only smartphone front camera data and machine learning algorithms to detect the position of body segments during exercise in real time in order to provide personalized feedback.

The aim of this study was to evaluate the ability of Motion Coach to detect and correct form during physiotherapeutic exercises in patients with osteoarthritis. We hypothesized that interrater agreement between physiotherapists and Motion Coach would be noninferior to that between 2 physiotherapists.

## Methods

### Participants

Participants with a confirmed prior diagnosis of osteoarthritis of the hip or knee were enrolled from the outpatient population of the Department of Orthopedics, Physical Medicine and Rehabilitation, University Hospital, Ludwig Maximilians University of Munich.

Inclusion criteria were (1) diagnosed hip or knee osteoarthritis and (2) age over 18 years. Exclusion criteria were (1) inability to consent (significant cognitive deficits); (2) not fluent in the German language; (3) severe medical or neurological conditions; (4) severe joint contractures that would influence the correct execution of the exercises; (5) previous hip, knee, and ankle arthrodesis; (6) osseous instabilities; or (7) severe osteoporosis.

### Ethics and Registration

The study was approved by the Ethics Committee of Ludwig Maximilians University of Munich (20-162) and all participants provided informed consent before study procedures were carried out. The study was registered with the German Study Registry (*Deutsches Register Klinischer Studien*; DRKS00021828) prior to beginning enrollment.

### Procedure

To evaluate the correction of osteoarthritis-specific exercises, Motion Coach provides instructions visually through an iPad’s screen and acoustically via headphones to the participants. While participants performed exercises using Motion Coach, 2 physiotherapists evaluated whether the exercises were being performed correctly. (Physiotherapists were blinded to the audiovisual feedback of Motion Coach). Furthermore, the physiotherapists evaluated the execution of an exercise set or the performance over the predefined time for static exercises as a whole on a 6-point Likert scale (0=insufficient, 5=excellent execution of movement).

### Exercises

**Figure 1:**
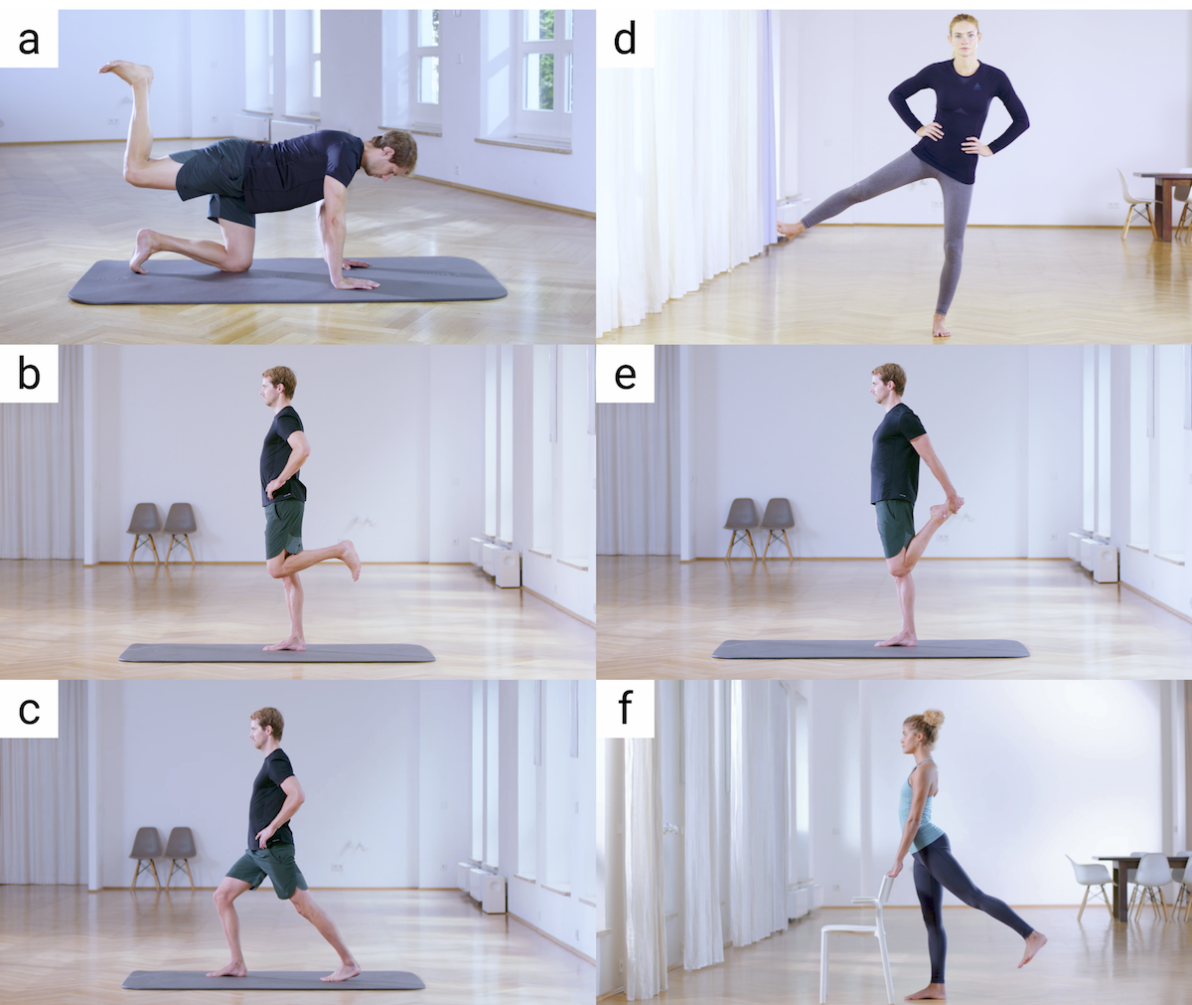
Exercises performed in this study (a) hip extension bent leg; (b) knee flexion (leg curl); (c) strengthening hip extensors; (d) strengthen hip abductors; (e) strain front of thigh; (f) elongation of the hip flexors.

For assessment, 6 exercises (Table 1 and Figure 1) that reflected several aspects of therapeutic exercises were chosen from the app to ensure detection by the algorithm was reliable in different circumstances. We included exercises that required a varying range of technical ability; exercises that had different modes of execution (4 dynamic and 2 static), to differentiate between exercises requiring rapid feedback in real time (due to continuous movement) and those that do not; and exercises with different levels of difficulty (low, medium, or high).

**Table 1:** Exercises performed by participants. Exercise difficulty was rated by training experts prior to study.

Label^a^	Exercise name	Pose	Execution mode	Exercise difficulty rating
a	Hip extension bent leg	Quadruped	Dynamic	High
b	Knee flexion (leg curl)	Standing	Dynamic	High
c	Strengthening hip extensors	Standing	Dynamic	Medium
d	Strengthen hip abductors	Standing	Dynamic	Medium
e	Strain front of thigh	Standing	Static	Medium
f	Elongation of the hip flexors	Standing	Static	Low

^a^Letters correspond to those in Figure 1.

### Motion Coach

#### Overview

**Figure 2:**
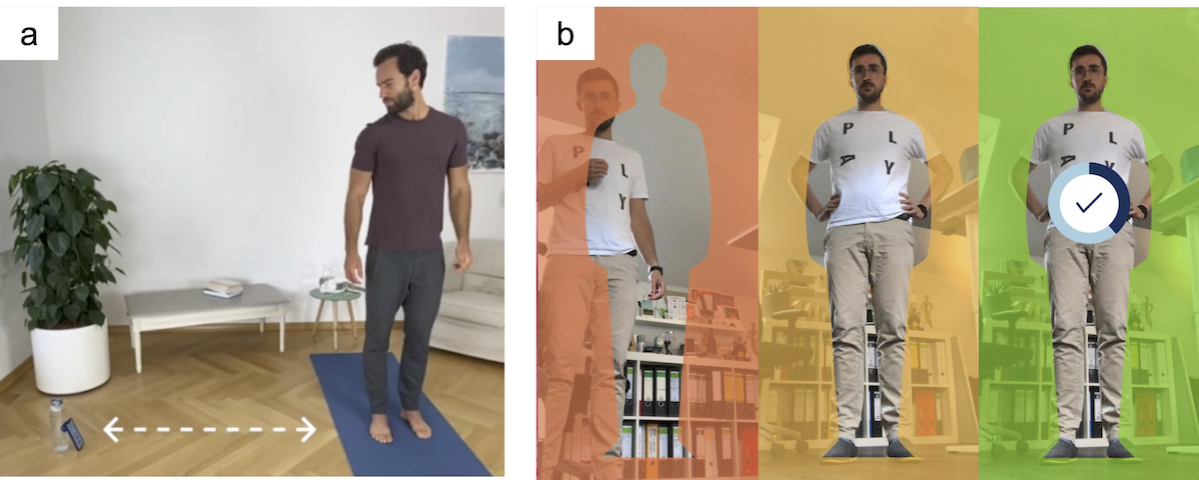
(a) User stands approximately 2 meters away from their device while the front-facing camera of the device captures user’s movements. (b) User is guided as to where to stand by a series of interactive screens.

In order to give audiovisual feedback on exercise form in real time, Motion Coach uses the camera stream of a user’s mobile device and artificial intelligence–based image processing. Users place their device on the ground approximately 2 meters away, tilted slightly so they can be seen in the frame of view of the camera. The app guides the user with interactive setup instructions (Figure 2). A 2-step process is applied to each new image frame as it is captured by the camera.

#### Step 1: Estimating Pose

**Figure 3:**
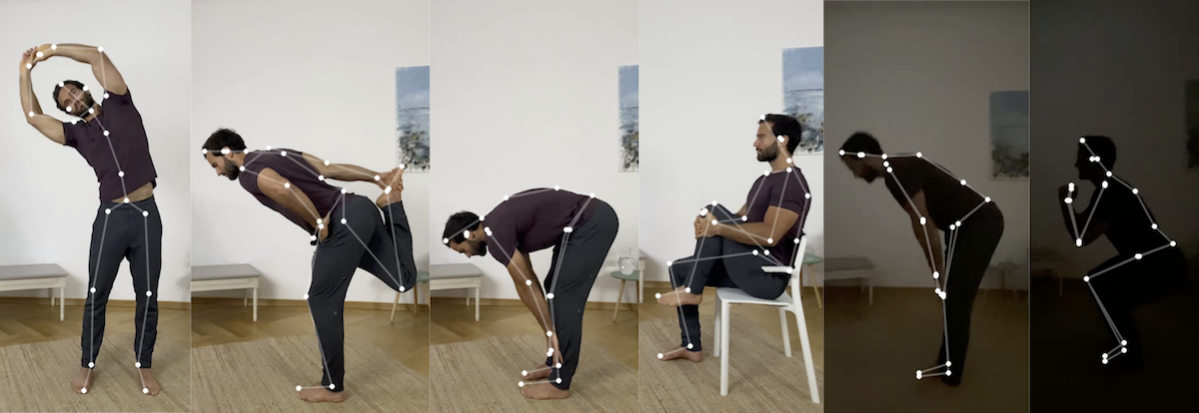
Examples of keypoint poses (white) inferred for various exercises by the Pose Estimation Model.

First, a Pose Estimation Machine Learning Model is applied to infer the user’s pose for each captured image frame in real time (Figure 3). This Pose Estimation Model is a convolutional neural network (typically used for image-based machine learning tasks [18]) with a proprietary architecture that runs entirely on the user’s mobile device (therefore, no raw video data leave the user’s device). The model was specifically optimized to run on a wide variety of iOS and Android devices, and the model achieves state-of-the-art performance on academic benchmarks such as the MPII Human Pose Data Set Benchmark [19]. Kaia Health trained this model using a proprietary image data set that consisted of data from people with a variety of characteristics (body shape, height, skin color, movement limitations, etc) exercising in front of their mobile device, with a wide variety of exercise movements and environmental conditions such as varying lighting and background to make the model robust. Each image in the data set had been manually labeled according to a taxonomy designed to best capture the human body in physiotherapeutic exercises.

#### Step 2: Evaluating Geometric Expert System

For audiovisual feedback, spatiotemporal constraints, which were configured in advance by medical, physiotherapeutic, or sport science–trained Kaia staff, are triggered based on movement; there was no need for reconfiguration on a per-user or per-session basis. While the system was in use, constraints were checked automatically in real time, and feedback was provided if any of the configured constraints were violated. If multiple constraints were violated, the prioritization mechanism selects the feedback based on risk of injury.

### Data

#### Overview

Physiotherapists’ evaluations were collected on a rating sheet for each participant. Data from the app were obtained by taking a screenshot of the report of corrections after the exercises had been executed. Baseline data were collected from participants using paper-based surveys or from participants’ medical reports if they were available in the system. Data from all sources were entered into a metafile in a spreadsheet (Excel; Microsoft Inc).

#### Data Collection

Gender, age, diagnosis, location of osteoarthritis, height, weight, and the Western Ontario and McMaster Universities Arthritis Index (WOMAC) score were collected at baseline [20].

Each participant performed 6 exercises with a total of 23 rated segments (a set of repetitions of 10 for each exercise or 30 seconds of stable posing for static exercises). For each segment, each physiotherapist’s evaluation and Motion Coach’s evaluation (ie, whether correction was required or not) were collected after the participants completed each exercise. Furthermore, the overall form rating by physiotherapists was recorded on a 6-point Likert scale. Data were pooled for the primary analysis.

### Study Endpoints

The primary endpoint was overall agreement between physiotherapists’ and Motion Coach’ evaluations during exercise execution. For each segment, there was a dichotomous outcome (correction recommended or not).

### Sample Size

We calculated the sample size required for a noninferiority trial with dichotomous outcome (ie, agreement or disagreement, either between app and physiotherapists’ ratings or between the 2 physiotherapists). We used pilot data (app–physiotherapists mean ratio 0.83; physiotherapist 1–physiotherapist 2 mean ratio 0.845) from the first 16 participants of the study. We determined that 552 exercise segments would be required; therefore, given an assumption of 23 segments per participant, the number of required participants was 24 (noninferiority margin 0.05; α=5%; β=90%). A noninferiority margin of 0.05 was recently used in a comparable study [16] for evaluation of exercise correction with a digital tool.

### Statistical Analysis

Continuous data (age, weight, height, and BMI) are described using means and standard deviations; discrete data (gender, location of osteoarthritis, WOMAC score) are described using absolute and relative numbers. Motion Coach–physiotherapist 1, Motion Coach–physiotherapist 2, Motion Coach–both physiotherapists, and physiotherapist 1–physiotherapist 2 interrater reliabilities (Cohen κ and percent agreement) were compared using *z* scores (α=5%). To assess whether demographic variables had any significant effect on the interrater agreement between Motion Coach and physiotherapists, subgroups for age (70 years or under, older than 70 years), gender (male, female), and BMI (30 kg/m^2^ or under, greater than 30 kg/m^2^) were formed and compared. We also assessed interrater agreement by exercise. Interrater agreement was categorized according to Cohen κ values as suggested by Landis and Koch [21]: κ < 0.00, poor agreement; κ=0.00-0.20, slight agreement; κ=0.21-0.40, fair agreement; κ=0.41-0.60, moderate agreement; κ=0.61-0.80, substantial agreement; κ=0.81-1.00, almost perfect agreement. All analyses were conducted with R software (version 4.0.2; R Foundation for Statistical Computing).

## Results

### Participants

The study population’s mean age was 67.6 (SD 8.98 years), and 20 out of the 24 participants (83%) were female. Participants (Table 2) had osteoarthritis of the knee (15/24, 62.5%), hip (6/24, 25%), or both knee and hip (3/24, 12.5%).

The mean global WOMAC score was 64.9 (SD 43.3) with mean domain scores of 15.8 (SD 10.7) for Pain, 7.3 (SD 4.8) for Stiffness, and 41.9 (SD 30.5) for Physical Function.

**Table 2:** Study population characteristics.

Characteristic				Value (n=24)
**Gender, n (%)**				
	Male			4 (17)
	Female			20 (83)
**Age (years)**				
	mean (SD)			67.6 (9.0)
	**n (%)**			
		≤70	12 (50)	
		>70 years	12 (50	
Weight (kg), mean (SD)				69.5 (16.7)
Height (m), mean (SD)				1.7 (0.1)
**BMI (kg/m^2^)**				
	mean (SD)			24.9 (4.6)
	**n (%)**			
		≤30 kg/m^2^	20	
		>30 kg/m^2^	4	
**Location of osteoarthritis, n (%)**				
	Hip			6 (25)
	Knee			15 (63)
	Both hip and knee			3 (13)
**WOMAC^a^, n (%)**				
	Total score			65 (43)
	Pain			16 (11)
	Stiffness			7 (5)
	Physical function			42 (31)

^a^WOMAC: Western Ontario and McMaster Universities Arthritis Index.

### Primary Analysis

Mean agreement between the app and physiotherapists (percent agreement 0.828) was not inferior (margin 0.05; *P*<.001) to that between physiotherapist 1 and physiotherapist 2 (percent agreement 0.833).

### Comparison of Interrater Reliability

**Figure 4:**
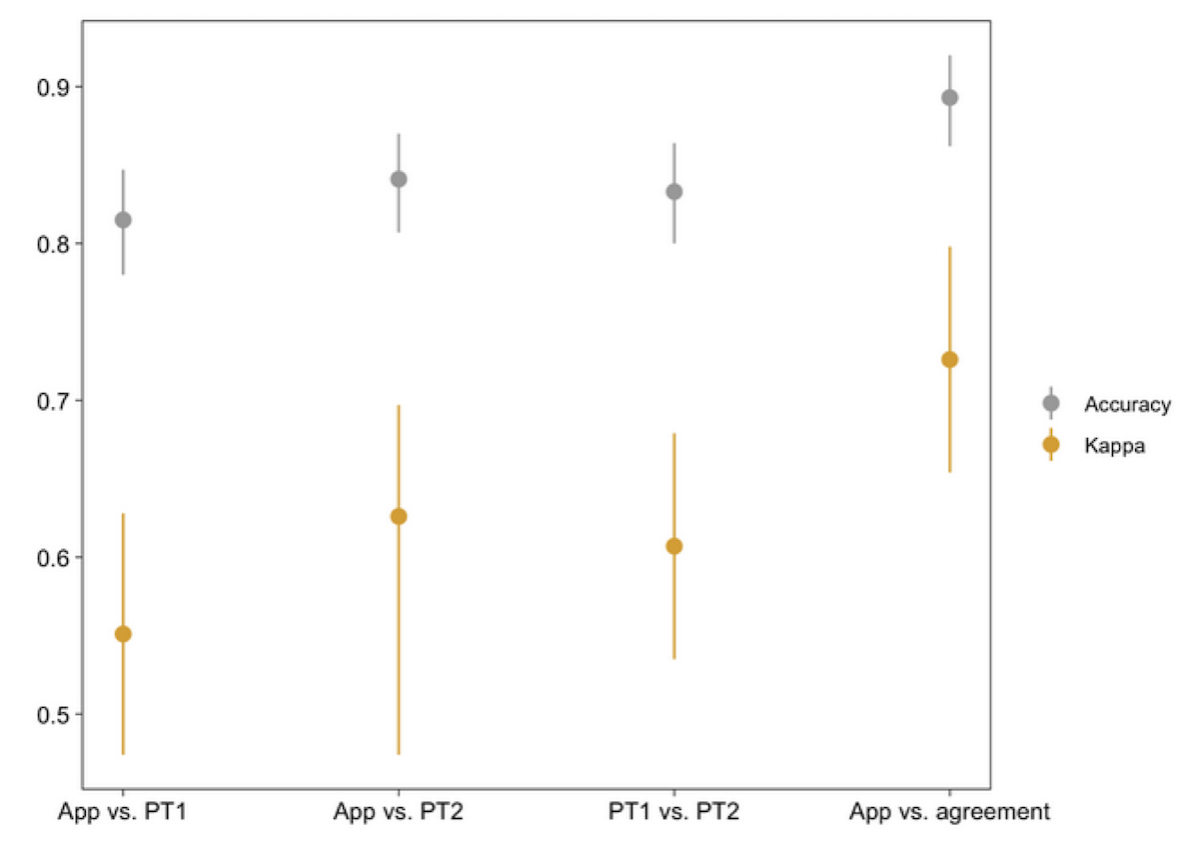
Interrater reliability (percent agreement and Cohen κ, with upper and lower 95% confidence intervals). PT: physiotherapist.

Interrater reliability for the evaluations (Table 3) demonstrated moderate to substantial agreement between physiotherapist 1 and physiotherapist 2 (Cohen κ=0.607, 95% CI 0.535-0.679; percent agreement 0.833, 95% CI 0.800-0.864), Motion Coach and physiotherapist 1 (Cohen κ=0.551, 95% CI 0.474-0.628; percent agreement 0.815, 95% CI 0.780-0.847); Motion Coach and physiotherapist 2 (Cohen κ=0.626, 95% CI 0.474-0.697; percent agreement 0.841, 95% CI 0.807-0.870); and Motion Coach and when there was agreement between both physiotherapists (Cohen κ=0.726, 95% CI 0.654-0.798; percent agreement 0.893, 95% CI 0.862-0.920) (Figure 3). There was no detectable difference between either Motion Coach–physiotherapist 1 interrater reliability and physiotherapist 1–physiotherapist 2 interrater reliability (Cohen κ: *P*=.309; percent agreement: *P*=.46) or Motion Coach–physiotherapist interrater reliability and physiotherapist 1–physiotherapist 2 interrater reliability (Cohen κ: *P*=.71; percent agreement: *P*=.74; Figure 4).

**Table 3:** All interpretations of correct versus incorrect exercise execution.

Assessment				All		Individual									Both					
					Physiotherapist 1					Physiotherapist 2				Agreement						Disagreement
					Correct		Incorrect		Correct		Incorrect		Correct			Incorrect				
**All, n**				552		394		158		374		178		338			122		92	
	**Physiotherapist 1, n (%)**																			
		Correct	394 (71.4)		N/A^a^		N/A		338 (90.4)		56 (31.5)		N/A			N/A		N/A		
		Incorrect	158 (28.6)		N/A		N/A		36 (9.6)		122 (68.5)		N/A			N/A		N/A		
	**App, n (%)**																			
		Correct	390 (70.7)		341 (86.5)		49 (31.0)		338 (90.4)		52 (29.2)		314 (92.9)			25 (20.5)		51 (55.4)		
		Incorrect	162 (29.3)		53 (13.5)		109 (69.0)		36 (9.6)		126 (70.8)		24 (7.1)			97 (79.5)		41 (44.6)		

^a^N/A: not applicable.

### Subgroup Analysis

**Figure 5:**
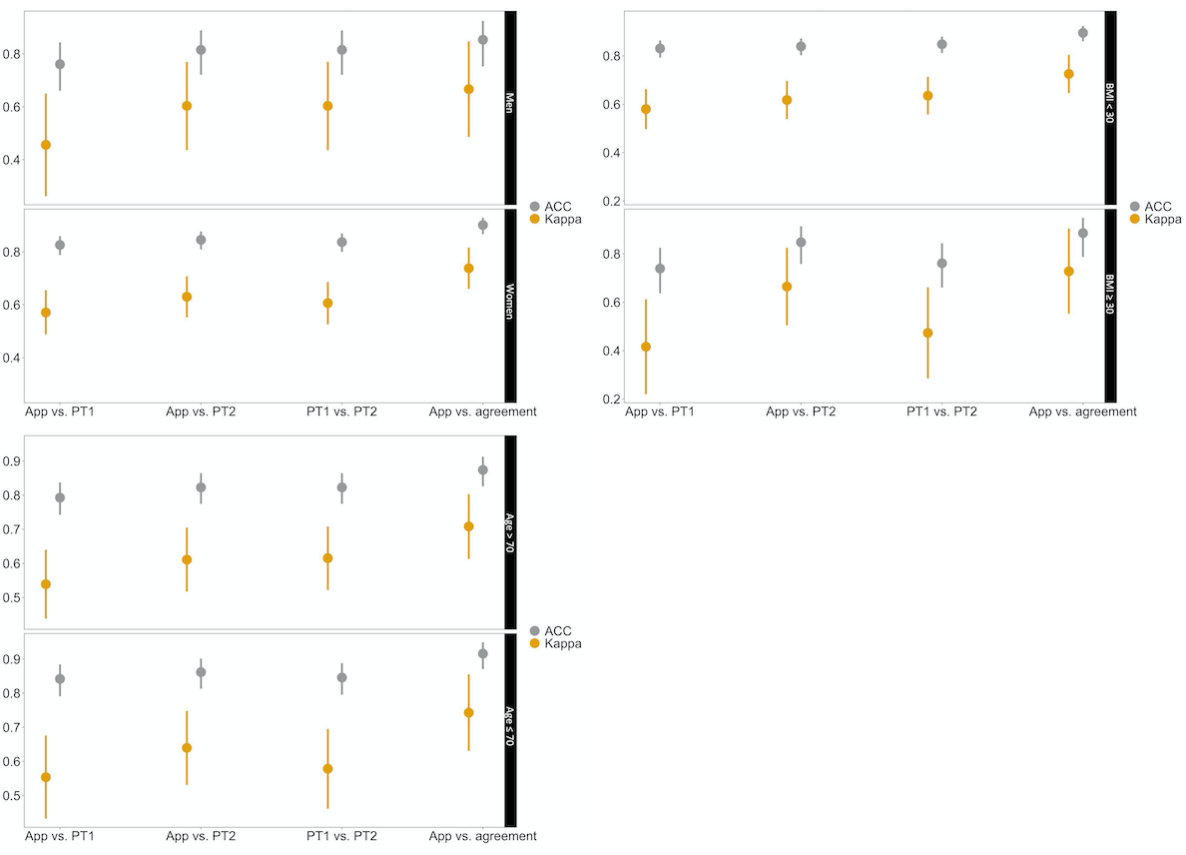
Interrater reliability (percent agreement and Cohen κ, with upper and lower 95% confidence intervals) for (a) gender, (b) BMI, and (c) age subanalyses. PT: physiotherapist.

No differences were found between app–physiotherapist interrater reliabilities and physiotherapist 1–physiotherapist 2 interrater reliability in any of the subgroups (Table 4 and Figure 5).

**Table 4:** Interrater agreement for age, gender, and BMI subgroups.

Raters						Cohen κ							Percent agreement			
				Mean (95% CI)			*P* value^a^			Mean (95% CI)				*P* value^a^		
**All**																
	**Physiotherapist 1 vs physiotherapist 2**				0.607 (0.535-0.679)						0.833 (0.800-0.863)					
			App vs physiotherapist 1	0.551 (0.474-0.628)			.31			0.815 (0.780-0.847)				.46		
			App vs physiotherapist 2	0.626 (0.556-0.697)			.71			0.841 (0.807-0.870)				.74		
	App vs agreement				0.726 (0.654-0.798)						0.893 (0.862-0.920)					
**Gender**																
	**Male**															
		**Physiotherapist 1 vs physiotherapist 2**			0.603 (0.436-0.770)						0.815 (0.721-0.889)					
			App vs physiotherapist 1	0.456 (0.262-0.650)			.26			0.761 (0.661-0.844)				.34		
			App vs physiotherapist 2	0.603 (0.436-0.770)			>.999			0.815 (0.721-0.889)				>.999		
		App vs agreement			0.667 (0.486-0.847)						0.853 (0.753-0.924)					
	**Female**															
		**Physiotherapist 1 vs physiotherapist 2**			0.606 (0.526-0.686)						0.837 (0.800-0.870)					
			App vs physiotherapist 1	0.571 (0.488-0.655)			.55			0.826 (0.788-0.860)				.65		
			App vs physiotherapist 2	0.630 (0.552-0.708)			.67			0.846 (0.809-0.877)				.71		
		App vs agreement			0.738 (0.660-0.816)						0.901 (0.867-0.929)					
**BMI**																
	**<30 kg/m^2^**															
		**Physiotherapist 1 vs physiotherapist 2**			0.635 (0.558-0.713)						0.848 (0.812-0.879)					
			App vs physiotherapist 1	0.580 (0.497-0.663)			.34			0.830 (0.793-0.864)				.46		
			App vs physiotherapist 2	0.617 (0.539-0.696)			.75			0.839 (0.802-0.872)				.71		
		App vs agreement			0.725 (0.646-0.804)						0.895 (0.860-0.923)					
	≥**30 kg/m^2^**															
		**Physiotherapist 1 vs physiotherapist 2**			0.473 (0.285-0.662)						0.761 (0.661-0.844)					
			App vs physiotherapist 1	0.416 (0.220-0.612)			.68			0.739 (0.637-0.825)				.72		
			App vs physiotherapist 2	0.665 (0.504-0.825)			.13			0.848 (0.758-0.914)				.11		
		App vs agreement			0.728 (0.552-0.904)						0.886 (0.787-0.949)					
**Age**																
	≤**70 years**															
		**Physiotherapist 1 vs physiotherapist 2**			0.578 (0.461-0.695)						0.846 (0.795-0.888)					
			App vs physiotherapist 1	0.554 (0.432-0.676)			.77			0.842 (0.791-0.885)				.90		
			App vs physiotherapist 2	0.640 (0.531-0.748)			.45			0.862 (0.813-0.902)				.59		
		App vs agreement			0.743 (0.631-0.855)						0.916 (0.870-0.949)					
	**>70 years**															
		**Physiotherapist 1 vs physiotherapist 2**			0.615 (0.522-0.708)						0.823 (0.775-0.864)					
			App vs physiotherapist 1	0.539 (0.438-0.640)			.28			0.793 (0.742-0.837)				.33		
			App vs physiotherapist 2	0.611 (0.517-0.705)			.95			0.823 (0.775-0.864)				>.999		
		App vs agreement			0.708 (0.613-0.803)						0.874 (0.826-0.913)					

^a^Comparison of subrow with *Physiotherapist 1 vs physiotherapist 2.*

### Interrater Agreement in Different Exercises

The analysis showed no detectable difference in the rates of interrater agreement in any of the exercises (Table 5 and Table 6).

**Table 5:** Mean rating of exercise form by the physiotherapists, using a 6-point Likert scale, and interrater agreement comparisons between app–physiotherapist and physiotherapist 1–physiotherapist 2 percent agreement values for each exercise.

Exercise^a^	Rating, mean (SD)	Percent agreement					*P* value	
		App–physiotherapist 1	App–physiotherapist 2	Physiotherapist 1–physiotherapist 2	App–agreement	Comparison 1^b^		Comparison 2^c^
a	2.8 (1.1)	0.804 (0.735-0.861)	0.845 (0.782-0.896)	0.851 (0.788-0.896)	0.881 (0.816-0.929)	.32		.23
b	3.4 (1.3)	0.792 (0.680-0.878)	0.806 (0.695-0.889)	0.875 (0.776-0.889)	0.841 (0.727-0.921)	.86		.13
c	4.3 (1.4)	0.778 (0.664-0.867)	0.750 (0.634-0.845)	0.750 (0.634-0.845)	0.852 (0.729-0.934)	.67		.67
d	4.5 (1.1)	0.889 (0.793-0.951)	0.889 (0.793-0.951)	0.889 (0.793-0.951)	0.938 (0.840-0.983)	>.999		>.999
e	4.5 (1.2)	0.833 (0.727-0.911)	0.861 (0.759-0.931)	0.778 (0.664-0.931)	0.946 (0.851-0.989)	.48		.36
f	4.8 (1.0)	0.812 (0.720-0.885)	0.875 (0.792-0.934)	0.833 (0.744-0.934)	0.912 (0.828-0.964)	.12		.68

^a^Letters correspond to those in Figure 1.

^b^App–physiotherapist 1 vs physiotherapist 1–physiotherapist 2.

^c^App–physiotherapist 2 vs physiotherapist 1–physiotherapist 2.

**Table 6:** Mean rating of exercise form by physiotherapists, using a 6-point Likert scale, and comparisons between app–physiotherapist and physiotherapist 1–physiotherapist 2 Cohen κ values for each exercise.

Exercise^a^	Rating, mean (SD)	Cohen κ					*P* value	
		App–physiotherapist 1	App–physiotherapist 2	Physiotherapist 1–physiotherapist 2	App–agreement	Comparison 1^b^		Comparison 2^c^
a	2.8 (1.1)	0.534 (0.396-0.673)	0.655 (0.396-0.673)	0.656 (0.396-0.673)	.707 (0.396-0.673)	.20		.20
b	3.4 (1.3)	0.579 (0.391-0.766)	0.605 (0.391-0.766)	0.749 (0.391-0.766)	.679 (0.391-0.766)	.85		.17
c	4.3 (1.4)	0.464 (0.242-0.687)	0.397 (0.242-0.687)	0.425 (0.242-0.687)	.596 (0.242-0.687)	.68		.81
d	4.5 (1.1)	0.680 (0.476-0.884)	0.695 (0.476-0.884)	0.714 (0.476-0.884)	.817 (0.476-0.884)	.92		.81
e	4.5 (1.2)	0.597 (0.393-0.801)	0.681 (0.393-0.801)	0.478 (0.393-0.801)	.867 (0.393-0.801)	.55		.44
f	4.8 (1.0)	0.435 (0.218-0.651)	0.635 (0.218-0.651)	0.453 (0.218-0.651)	.616 (0.218-0.651)	.17		.91

^a^Letters correspond to those in Figure 1.

^b^App–physiotherapist 1 vs physiotherapist 1–physiotherapist 2.

^c^App–physiotherapist 2 vs physiotherapist 1–physiotherapist 2.

## Discussion

The purpose of this study was to compare interrater agreement of osteoarthritis knee and hip exercise assessments between Motion Coach (a novel digital tool) and trained physiotherapists; we hypothesized that assessment agreement for the Motion Coach app would not be inferior to that of physiotherapists. Our data support the hypothesis that Motion Coach is noninferior to physiotherapists in assessing whether exercise poses required correction. There was no difference between the interrater agreement of Motion Coach and physiotherapists and that among physiotherapists. This finding was also true in analyses of subgroups that consisted of men, women, participants 70 years or older, participants below 70 years, participants with BMI greater than 30 kg/m^2^, and participants with BMI less than 30 kg/m^2^ and in analyses by exercise. To the best of our knowledge, this is the first report comparing a digital software–based exercise feedback tool with conventional smartphone technology and physiotherapeutic exercise feedback for musculoskeletal conditions.

Previous studies [16,17] have used 3D sensors such as the Microsoft Kinect system to assess pose during exercise and give feedback to users if correction was needed. However, 3D-sensor systems are expensive and require extensive external hardware and a stationary television set, and thus have limited scalability in providing access to digital rehabilitation. Komatireddy [16] found no detectable difference in agreement between a software solution for Microsoft Kinect and a panel of physiotherapists for repetition count and the number of acceptable exercises. Wochartz et al [17] evaluated agreement with regard to joint angles and positions of the lower limb between a Microsoft Kinect based-system and a 3D camera-based motion system but did not evaluate its capacity to trigger corrections during therapeutic exercises; they concluded that the validity of the Kinect system to detect pose without postprocessing was restricted.

Other digital rehabilitation tools for musculoskeletal pain use external inertial sensors attached to specific limbs or joints to detect exercise poses [22-24]. By nature, these systems are limited to detecting the poses of joints or body areas only where they are placed, and users must typically attach the hardware to their bodies themselves. Studies [14,15] have shown that these systems are generally capable of detecting exercise poses; however, these systems have not been systematically evaluated for their ability to provide feedback on pose during exercise execution.

Built-in smartphone inertia sensors are a viable option to deliver pose correction in rehabilitation without requiring specialized equipment or installations. Spina et al evaluated real-time smartphone motion sensor data processing as an option to assess pose in physical exercises by people with chronic obstructive pulmonary disease [25]. The system was able to provide feedback on pose and exercise feedback similar to the feedback of a trained therapist. The system required a holster to hold the smartphone and that was repositioned on the body depending on the exercise performed. While previous reports have addressed the general feasibility of exercise-related feedback using 2D RGB camera streams, the percent agreement of those systems without postprocessing limited their use [26,27]. In contrast, Motion Coach relies upon 2D camera stream postprocessing of using machine learning algorithms for valid real-time feedback for exercise correction.

To the best of our knowledge, this study is the first to evaluate the potential of a technology (Motion Coach) to trigger suitable corrections of therapeutic exercises in musculoskeletal pain rehabilitation, with the findings suggesting that Motion Coach technology triggers valid corrections as compared to trained physiotherapists. Motion Coach is a software only solution operating on off-the-shelf smartphones, without any need for additional hardware, which makes this digital therapeutic solution accessible to a broad patient population.

The interrater reliability of trained physiotherapists assessments of pose during lower extremity exercises for the has been investigated: Chmielewski et al [28] investigated interrater agreement during 2 exercises performed by healthy volunteers for the lower extremity with 2 distinct methods (overall rating and investigation of deviation from the neutral plane during exercise) in a panel of 3 physiotherapists and found agreement better than chance but no high levels of agreement between physiotherapists. Whatman et al [29] investigated interrater agreement for lower extremity exercises in a panel of physiotherapists (segment-specific and overall agreement) with ordinal and dichotomous outcomes; interrater agreement was generally fair to good and increased with experience of the rater. The interrater agreement observed in our study, among the physiotherapists and also between the physiotherapists and Motion Coach, was high compared to those in previous studies [28,29]. This finding can be explained by the high level of experience of the physiotherapists and training of the physiotherapists on evaluation criteria prior to patient enrollment. Compared to other approaches requiring specialized hardware, the degree of agreement between both physiotherapists and Motion Coach remains high; a similar study [30] using data from the Kinect version 2 Skeleton Tracking system to assess rehabilitation exercises in 19 people with musculoskeletal and neurological limitations showed a limited correlation (*r*=0.60, *P*<.01 for the clinical subgroup) between expert’s clinical judgement and the results of various models based on sensor data.

The study had several limitations. First, the pool of raters was small with n=2, and a third rater was not used (in cases of disagreement between the 2 raters). In addition, the sample was heterogeneous in terms of gender distribution and localization of osteoarthritis, limiting the generalizability of the results. Other limitations arise from the fact that the assessment of pose during therapeutic exercise execution is not standardized, and thus, in this study as in comparable previous studies [28,29], no well-established standard measurement could be used to quantify exercise execution. Furthermore, dichotomous assessment of acceptable exercise is only one of several measures used in prior studies to assess form during exercise. Future studies evaluating Motion Coach will need to use more diverse outcome measures of form during exercise, for example calculations with a musculoskeletal human model.

The interrater agreement for suggesting corrections during therapeutic exercises between both physiotherapists and Motion Coach was moderate to substantial and did not differ between physiotherapists themselves and physiotherapists and Motion Coach. This finding was valid for all investigated exercises and subgroup analysis. These findings validate the ability of Motion Coach to detect form during exercise and provide audiovisual feedback to users with preexisting musculoskeletal conditions.

## Abbreviations

WOMAC: Western Ontario and McMaster Universities Arthritis Index
